# Diversity and predictive metabolic pathways of the prokaryotic microbial community along a groundwater salinity gradient of the Pearl River Delta, China

**DOI:** 10.1038/s41598-018-35350-2

**Published:** 2018-11-23

**Authors:** Shilei Sang, Xiaoying Zhang, Heng Dai, Bill X. Hu, Hao Ou, Liwei Sun

**Affiliations:** 1 Department of Ecology, Jinan University, Guangzhou, 510632 Guangdong, China; 2 Institute of Groundwater and Earth Sciences, Jinan University, Guangzhou, 510632 Guangdong, China

## Abstract

Almost half of the groundwater in the Pearl River Delta (PRD) contains salt water originally derived from paleo-seawater due to the Holocene transgression, which then generates intense physicochemical gradients in the mixing zone between freshwater and saltwater. Although some studies have been conducted on the hydrological and geochemical characteristics of groundwater in the PRD to monitor the intrusion of seawater, little attention has been paid to the microbial community of this particular region. In this study, we implemented a high-throughput sequencing analysis to characterize the microbial communities along a salinity gradient in the PRD aquifer, China. Our results indicated that the microbial community composition varied significantly depending on the salinity of the aquifer. The presence of abundant anaerobic microorganisms of the genera *Desulfovibrio* and *Methanococcus* in certain saltwater samples may be responsible for the gas generation of H_2_S and CH_4_ in the stratum. In saline water samples (TDS > 10 g/L), the linear discriminant analysis effect size (LEfSe) analysis found two biomarkers that usually live in marine environments, and the aquifers of the PRD still contained large quantity of saltwater, indicating that the impact of the paleo-seawater has lasted to this day. The predictive metagenomic analysis revealed that the metabolic pathways present in the groundwater samples studied, included the degradation of pesticides and refractory organics (dichlorodiphenyltrichloroethane (DDT), atrazine and polycyclic aromatic hydrocarbons), matter cycling (methane, nitrogen and sulfur), and inorganic ion and mineral metabolites. This study can help enhance our understanding of the composition of the microbial assemblages and its implications as an environmental indicator in an aquifer affected by saltwater intrusion.

## Introduction

In China, groundwater is the most valuable water resource, and supplies water for nearly 70% of the total population and 40% of agricultural irrigation^1^. However, the rapid socio-economic development in the last few decades has increased the emission of various pollutants and created a shortage of fresh groundwater^2^, caused by the continuous increase in freshwater demand, the over-exploitation of groundwater and the discharge of domestic and industrial wastewater. Microorganisms are almost the only inhabitants in the groundwater environment^3^, and they have an important role in cycling nutrients and constituents through their metabolic activity^4^. Recent research suggests that an aquifer is an ecological system affected by the activity of microbial communities^5,6^, which is closely relate to nutrient transport, geochemical cycles and the degradation of pollutants^7,8^.

In general, microorganisms in aquifer ecosystems are sensitive to environmental changes and thus can be a useful indicator to biomonitor pollutants^9^. It is generally known that groundwater is more stable and cleaner compared to surface water, which occurs because of the filtration process through the aquifer material (i.e., gravel, silt, sand, clay) that leads to natural attenuation processes in the groundwater, such as dissolution and adsorption, oxidation-reduction reactions, and biochemical reactions^10^. However, the distribution and activity of microbial communities are actively influenced by various aquifer conditions^11,12^, such as geological setting, water quality, aquifer materials, and the type of aquifer. Smith *et al*.^13^ compared the composition of microbial assemblages inhabiting unconfined and confined aquifer ecosystem and found distinct variations in composition and metabolism driven by different nutrient inputs and aquifer types. Flynn *et al*.^14^ compared the community of microbes in different aquifer materials and concluded that the major populations differed between aquifer types. Thus, determining the characteristics of the groundwater microbial community, the microbial diversity and their potential functions will help to expand our understanding of a complex groundwater system and its related biogeochemical processes in another dimension.

An increasing number of studies have shown that the microbiome is an important part of the underground dynamic ecosystem and is an indispensable parameter to evaluate subsurface water quality^15,16^. Unlike surface water, such as the Mississippi River, that can contain as many as 3,107 to 5,498 species^17^, an aquifer is mostly oligotrophic, containing a small number of microorganisms. The presence of *E. coli* is typically used as an environmental indicator of fecal contamination^18^. Recently, other microbial parameters, including the microbial communities, diversity and interaction with physicochemical characterization, have been used as indicators to evaluate water quality^12,19^. For instance, to find biological parameters to monitor groundwater quality, Unno *et al*.^20^ analyzed the relationship between taxonomic groups and hydrological chemistry and found that microbiome analysis was an effective tool to monitor groundwater health. As expected, many different contaminants, which can be degraded by diverse microbes, have been found in aquifers. Ye *et al*.^21^ compared different microbial fingerprints with respect to sampling location in submarine groundwater, and selected some potential bacterial groups for bioremediation, such as *Comamonas spp*. To effectively understand and protect fresh groundwater, it is essential to investigate the composition and function of the microbial community in aquifers^13^.

The plain of the Pearl River Delta (PRD) in China is responsible for the water supply of 50 million people, but the groundwater is contaminated due to high salinity and pollution^22–24^, as well as continuous seawater intrusion into the Pearl River Estuary^25^. The groundwater salinity is relatively stable in most areas of the PRD, since it primarily originated from paleo-seawater^26,27^ due to the Holocene transgression^28^. In addition, groundwater in many areas of the southern part of the PRD has been found to contain more than 10 g/L of total dissolved solids (TDS)^29^. To improve the groundwater quality and management, numerous research focusing on the hydrological and geochemical characteristics of the PRD aquifers has been conducted^27,30^. However, few studies have investigated the microbial communities of the groundwater in the PRD.

To investigate the diversity and taxonomic composition of the microbial biocenosis in groundwater along a salinity gradient of the PRD, we used next generation sequencing (NGS) technology to establish the relationship between microbial communities and environmental variables from a hydrogeological point of view. Through the investigation of the spatial variation of the groundwater quality, microbial communities and functions in the PRD, the response of the underground microbial communities to the salinity gradient was obtained. Our study can help to monitor seawater intrusion and underground water quality from a biological perspective.

## Results and Discussion

### Hydrogeochemical and environmental parameters

Hydrogeochemical and environmental parameters are typically used to establish the salinity and origins of groundwater salinization^31^. The major hydrogeochemical parameters of the groundwater samples analyzed are listed in Table 1. According to the Schukalev classification method^32^, the primary chemical type of saltwater was Cl-Na, while the freshwater contained HCO3·(Cl)-Ca·Na, HCO3·Cl-Mg·Na or HCO3-Mg·Ca, which indicated that the groundwater underwent salinization derived from the paleo-seawater^26,27^. The parameter of the TDS was usually used to quantify groundwater salinity based on the following criteria^33^: non-saline/freshwater (F) (0 < TDS < 1 g/L), brackish water (B) (1 < TDS < 10 g/L) and saline water (S) (10 < TDS < 100 g/L). Based on this classification, the 12 monitoring wells were divided into four saline samples (S), four brackish samples (B) and four freshwater samples (F).

**Table 1:** Hydrogeochemical and environmental parameters of the 12 groundwater samples.

Sample	Q149	Q141	Q137	Q146	Q138	Q140	Q144	Q143	Q130	Q132	Q124	Q135
T (°C)	26.0	25.2	24.0	25.1	24.9	23.0	25.1	25.0	25.8	24.5	25.0	22.7
pH	7.68	6.46	6.6	7.04	6.53	5.82	7.09	6.97	6.88	7.26	7.37	7.82
ORP	−220.1	9.3	−84.4	−100.7	−29.6	−104.1	32.5	29.1	71.2	−30.6	45.5	−98.2
DO (%)	1.4	41.2	27.1	28.8	26.4	13.3	55.2	49	28.5	60.8	48.2	25
salinity	19.48	18.41	17.9	13.27	5.97	5.5	4.1	4.0	0.81	0.46	0.27	0.15
TN (mg/L)	87.27	3.09	1	1.27	4.88	2.77	3.78	1.37	47.59	32.42	0.93	25.69
TP (umol/L)	0.8	0.92	0.92	1.04	0.56	0.5	0.56	0.68	0.56	2.58	0.5	0.32
TDS (g/L)	15.3	14.52	14.17	10.79	5.136	4.77	3.61	3.52	0.74	0.41	0.22	0.10
EC (ms/cm)	31.22	29.6	28.92	22.02	10.48	9.741	7.35	7.19	1.42	0.84	0.76	0.19
TOC (mg/L)	3.08	3.46	7.41	2.85	3.43	53.71	2.01	0.53	0.30	3.53	2.25	1.30
HCO_3_^−^ (mmol/L)	0.26	2.15	10.03	0.18	1.81	22.81	2.27	7.98	9.39	6.40	3.88	2.39
NO_3_^−^ (mg/L)	0.01	0.05	0.11	0.03	1.49	0.07	0.07	0.06	0.66	0.01	0.01	0.01
SO_4_^2−^ (mg/L)	57	1292	27.2	137	191.9	18.6	1.4	337.5	1.19	2.46	23.19	0.62
NO_2_^−^ (μg/L)	2.33	3.21	3.87	7.25	8.28	7.62	6.44	6.74	28.43	27.69	6.74	1.37
K^+^ (mg/L)	121.59	227.2	58.67	65.27	23.43	25.62	36.34	48.36	8.01	15.6	5.43	0.95
Ca^2+^ (mg/L)	849.48	542.7	889.82	1754.1	1311.1	1905.3	831.53	286.51	158.94	47.68	60.62	13.97
Mg^2+^ (mg/L)	539.06	553.59	452.42	382.72	265.67	238.07	127.44	128.48	37.29	32.65	8.56	13.29
Na^+^ (mg/L)	2456.1	2415.7	2321.3	2060.7	1237.8	894.49	538.25	1252.3	158.35	104.87	57.7	5.17
Cl^−^ (g/L)	13.44	11.37	12.36	9.45	4.46	3.55	2.49	2.09	0.23	0.09	0.02	0.006
Hydrochemical type	Cl-Na	Cl-Na	Cl-Na	Cl-Ca·Na	Cl-Ca·Na	Cl-Ca·Na	Cl-Ca·Na	Cl-Na	HCO_3_·Cl-Ca·Na	HCO_3_·Cl-Na·Mg	HCO_3_-Ca·Na	HCO_3_-Mg·Ca

### Alpha and beta diversity of the microbial community

In this study, a total of 448,645 sequences (294 bp average length) were obtained from twelve groundwater samples (including four saline monitoring wells (S) (TDS > 10 g/L), four brackish monitoring wells (B) (1 < TDS < 10 g/L)) and four freshwater samples (F) (TDS < 1 g/L)) generated in a single run on a HiSeq 2500 high-throughput sequencing system. For all 12 groundwater samples, the raw sequence reads ranged from 29,355 to 44,283. The lowest sequence number 29,355 was used to subsample all of the samples at the same level. After clustering and alignment, a total of 984 microbial OTUs were obtained based on a 97% threshold with a range of 364–634 OTUs (Table S2), indicating a remarkable variation of the microbial OTU number among the sampling sites. The lowest OTU number was found in the brackish sample Q140 (B), and the highest OTU number was also in the saltwater sample Q141 (S). Among all the 984 OTUs, some OTUs were only found in either the saltwater samples (n = 295, 29.98%) or the freshwater samples (n = 60, 6.10%), while most OTUs (n = 629, 63.92%) were shared by salt and fresh groundwater samples (Supplementary Fig. S1), indicating a potential exchange between the saltwater originating from paleo-seawater^26,27^ and the freshwater recharged from the surface water and precipitation^26^.

The microbial Chao richness estimator varied between 496 and 785 and the Heip’s evenness estimator was between 0.02 and 0.08 (Supplementary Table S1). Microbial richness and evenness, reflected by the Chao and Heip indices, varied along the salinity gradient but not significantly (P > 0.05) (Supplementary Table S2). As demonstrated by the Shannon index, the diversity of the microbial communities did not vary significantly along the salinity gradient of the aquifer in the PRD area, which was consistent with the study in the relatively stable estuary of the Baltic Sea, where no significant change in the Shannon diversity index was found along the salinity gradient^34^. However, an increase in salinity will result in a substantial change of the microbial diversity in a Bay estuary^35^ or aquifer^16^ where the environment is unstable. In the research which was conducted by Zhou *et al*.^16^, the bacterial Shannon diversity markedly changed from H′ = 3.22 ± 0.28 (autumn and winter) to H′ = 1.31 ± 0.35 (spring and summer) in the groundwater primarily due to the rise of the groundwater level, as well as the nutrient inputs. However, our research primarily compared the microbial diversity in aquifers of different salinity in the same period, and the environment was relatively stable. Hence, we concluded that there was no significant change in the microbial diversity in the groundwater studied in this research.

**Figure 1:**
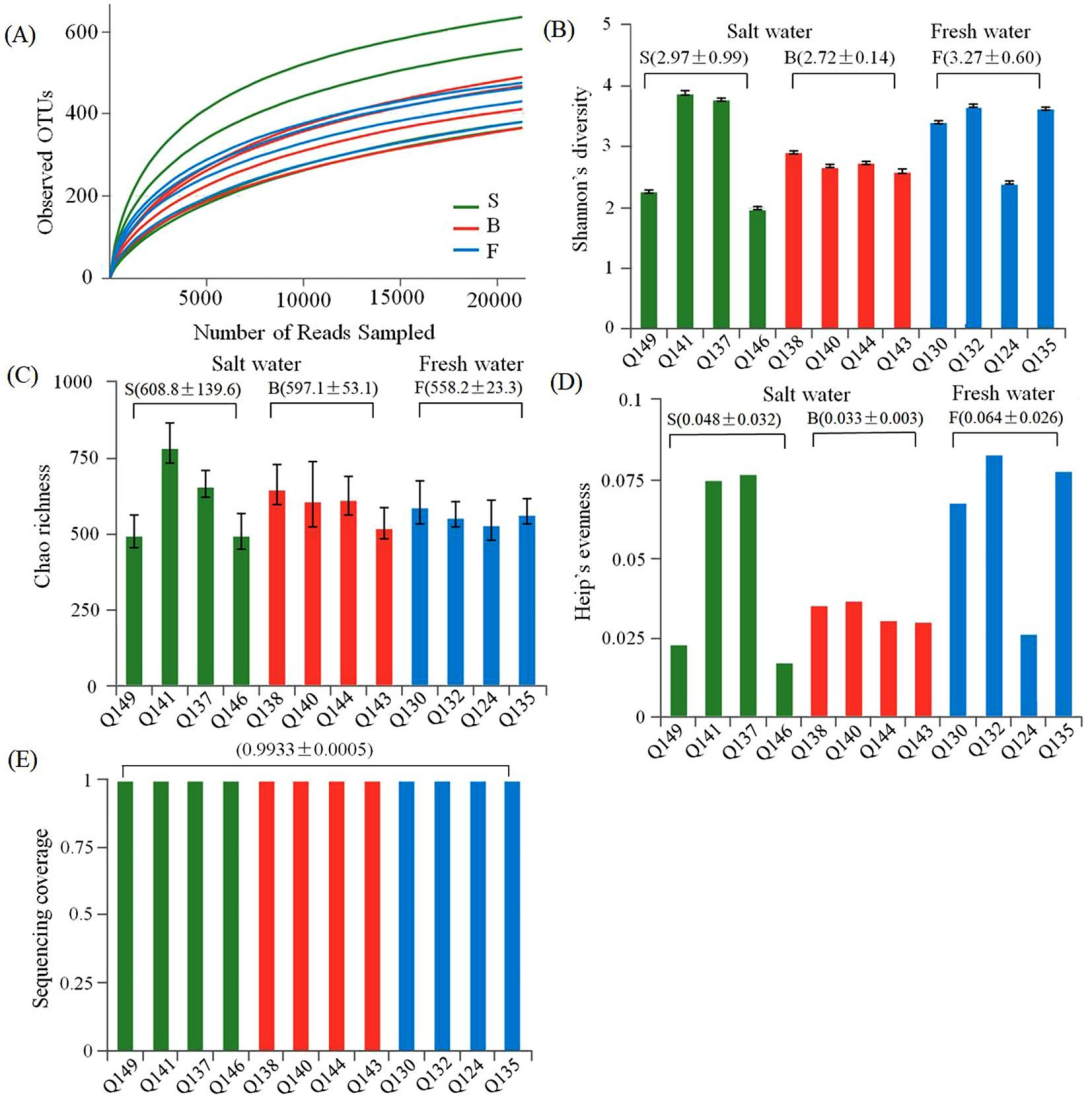
The calculated α-diversity indices of freshwater (F), brackish water (B) and saline water (S): (**A**) the rarefaction curves and the number of the observed OTUs. (**B**) Shannon’s diversity. (**C**) Chao richness. (**D**) Heip’s evenness. (**E**) Sequencing coverage. The numbers in parentheses are the mean and standard deviation. For more specific data of the α-diversity indices, please refer to Supplementary Table S1.

Surprisingly, the lowest diversity, richness and evenness were all found in a saltwater sample of Q146 (S), and the highest diversity and richness were also found in a saltwater sample of Q141 (S) (Supplementary Table S1). However, there was no significant difference for all the calculated alpha diversity indices, including the observed OTUs, Shannon’s diversity, Chao richness and Heip’s evenness (*P* > 0.05) between the samples, indicating that salinity may not be the most important factor determining microbial alpha diversity in groundwater (Table S4). Good’s coverage, which encompassed 99% of all the samples, reflected a perfect estimate of sampling completeness (Fig. 1E), which was consistent with the rarefaction curves that almost tended to reach an asymptote (Fig. 1A).

**Figure 2:**
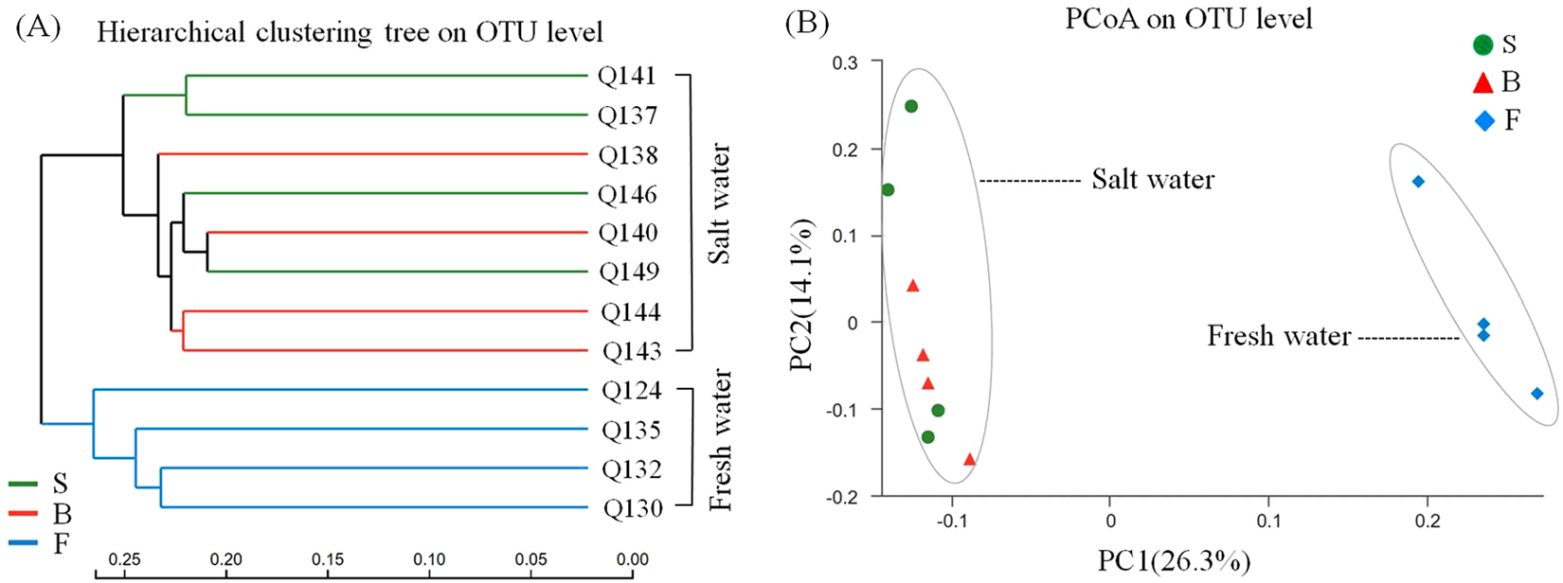
Beta diversity of saltwater and freshwater samples: (**A**) Hierarchical clustering tree on the OTU level based on the unweighted-unifrac distance. (**B**) Principal co-ordinates analysis (PCoA) on the OTU level based on the unweighted-unifrac distance.

In addition, the beta diversity of the groundwater samples was determined based on the unweighted-unifrac distance. According to the results of the hierarchical clustering tree at the OTU level, 12 samples were clustered into two groups, and it was clear that the saltwater samples tended to be distantly separated from freshwater samples (Fig. 2A). A principal co-ordinates analysis (PCoA) also demonstrated the remarkable variability of the groundwater samples along the salinity gradient, with the first axis (PC1) showing 26.3% of variation (Fig. 2B) and the second axis (PC2) of 14.1% reflected variation within the sampling site. The results suggested that the salinity gradient and spatial variability affected the microbial communities.

### Taxonomic composition of the microbial communities

The sequences were analyzed by the RDP Classifier algorithm against the Silva 16S rRNA database using a confidence threshold of 70% and clustered into different taxa including two domains, 44 phyla, 95 classes, 174 orders, 267 families and 420 genera. Not surprisingly, most of the sequences were identified as bacteria with an average percentage up to 95.09%, while the average percentage of the archaea was 4.91%. Although we used the primer of the V4 regions which were known to vastly improve the detection of species^36^, some of the sequences could still not be classified. The average percentage of the unclassified sequences increased with the refinement of the classification, ranging from 0.4 (phylum level) to 31.08% (genus level). In addition, the unclassified ratios of the saltwater samples were higher than those of the freshwater samples at the class, order and family levels, indicating that there may be more advanced sequences in the saltwater.

**Figure 3:**
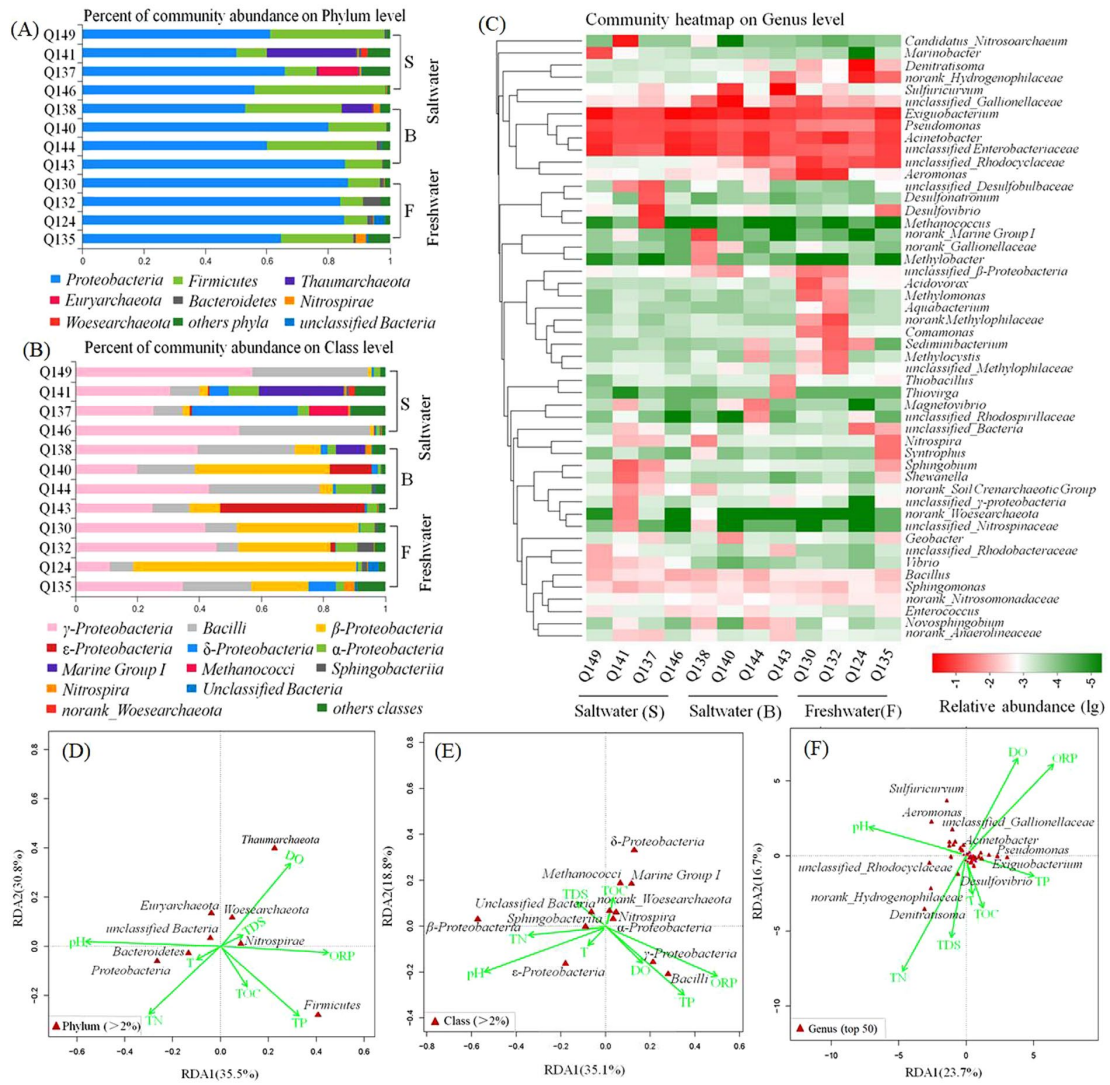
Microbial community composition of the groundwater samples studied (**A**) at the phylum level (relative abundance >2%), (**B**) at the class level (relative abundance >2%) and (**C**) at the genus level (top 50). (**D**–**F**) Show the RDA analysis of these microbial biocenoses and environmental parameters.

The taxonomic composition of the microbial communities at the phylum level (relative abundance >2% at least one sample) is shown in Fig. 3A (Supplementary Table S3). Consistent with earlier research^7,37^, a majority of the classified sequences were assigned to *Proteobacteria* (50.34–86.51%) in both the saltwater and freshwater samples. The second highest proportion was *Firmicutes*, which are able to produce spores to resist extreme conditions^38^, and a higher abundance of *Firmicutes* in the saltwater indicated that the saline groundwater environment was unfavorable for the growth of some microorganisms compared to the freshwater. The other dominant bacteria phylum was *Bacteroidetes* with the ability to degrade organic matter^39^, which was enriched in freshwater samples. The communities described in porous aquifers are primarily dominated by the members of different *Proteobacteria*, *Firmicutes*, *Actinobacteria* and *Bacteroidetes*^7^. Consistent with our results, the top three categories are primarily *Proteobacteria*, *Firmicutes* and *Bacteroidetes*. Hery^37^ studied the bacterial communities in a carbonate aquifer subject to seawater intrusion and found that the phylum with the highest abundance was *Proteobacteria* with a percentage of 58.8–93%. Consistent with our results, a majority of the classified sequences were assigned to *Proteobacteria* with a percentage of 50.34–86.51%. Other dominant phyla were also similar, including *Firmicutes*, *Bacteroidetes* and *Actinobacteria*. It is notable that the community structure of the archaea was dominated by *Thaumarchaeota*, *Euryarchaeota* and *Woesearchaeota*, which were significantly more abundant in saltwater than freshwater. Except for the eight major phyla, another 36 phyla with a relative abundance that was no more than 2% in any sample were classified as “other phyla” and included *Acidobacteria*, *Verrucomicrobia*, and *Actinobacteria*. In addition, the percentage of “other phyla” was between 0.78 and 8.89% with an average of 3.21%.

The composition of the microbial communities at the class level is compared further, as shown in Fig. 3B (Supplementary Table S4). Among the 12 representative classes with a relative abundance >2%, at least one sample, and as many as eight classes, were affiliated with *Proteobacteria*, *Firmicutes*, *Thaumarchaeota* and *Euryarchaeota*. In *Proteobacteria*, the *γ-proteobacteria* was dominant in most samples with an abundance of 11.01–52.87%, followed by *β-*, *ε-*, *δ-* and *α- proteobacteria*. It was obvious that *β-proteobacteria* was more abundant in freshwater than saltwater, except for the Q140 sample, which coincides with previous reports that *β-*proteobacteria was dominant in fresh water on a global scale^40,41^. Simultaneously, the classes *Marine Group I* and *Methanococci*, which were affiliated with *Thaumarchaeota* and *Euryarchaeota*, respectively, were primarily found in saltwater. In *Firmicutes*, the dominant class was *Bacilli* with a relatively higher percentage in saltwater than in freshwater. Except for the classes belong to the phyla *Proteobacteria*, *Firmicutes*, *Thaumarchaeota* and *Euryarchaeota*, other dominant classes were affiliated with *Nitrospirae*, *Woesearchaeota* and *Bacteroidetes*.

The hierarchical heatmap at the genus level (top 50) of the groundwater samples analyzed is presented in Fig. 3C (Supplementary Table S5). The freshwater samples had *Acidovorax, Aquabacterium, Denitratisoma* and *Comamonas* with a relatively high abundance, while the saltwater contained some genera related to carbon, nitrogen and sulfur cycles, such as *Methanococcus*, *Candidatus_Nitrosoarchaeum* and *Desulfovibrio*. The presence of abundant heterotrophic anaerobic microorganisms of the genera *Desulfovibrio* and *Methanococcus* validated the previous assumption that there was microbial SO_4_^2−^ reduction and methanogenesis happening in the PRD aquifer^26^. When hydrogeological surveys were conducted by drilling boreholes, the odor of rotten eggs was commonly smelled, indicating the emission of H_2_S gas. The presence of methanogens in the aquifer is confirmed by the existence of CH_4_ gas in the quaternary aquifer identified by the Guangdong Geological Survey^42^. The results indicate that alternative bacterial metabolic processes, such as SO_4_^2−^ reduction and methanogenesis, may be responsible for the gas generation of H_2_S and CH_4_ in the stratum. In addition, the suitable primers to amplify sulfate-reducing bacteria (SRB) and methanogenic archaea (MA) were also designed and synthesized (Supplementary Fig. S2). Their amplification with the primers used was satisfactory, which verified the results of the high-throughput sequencings and demonstrated that SRB and MA were present in the microbial community. In addition, some types of clones, which correlated with aerobic microbial groups, such as *Exiguobacterium*, *Pseudomonas* and *Acinetobacter*, were revealed in all the groundwater samples with a relatively high yield. In addition, 40 of the top 50 genera were present in all the groundwater samples, which may reflect the ecological coherence of municipal groundwater ecosystems^43^.

In addition, redundancy analysis (RDA) was performed on the microbial communities and the main environmental factors (Fig. 3D–F). At the phylum level, acute angles emerged among the DO, TDS and ORP, indicating a synergistic effect on the microbial community, which suggested an opposite effect of the pH, temperature and total nitrogen (TN). However, at the class and genus level, the DO and ORP both exhibited an opposite effect on the TDS, indicating that the relationship between the environmental variables and microbial communities in groundwater ecosystem is complex^34,44^. We can therefore conclude that the structure of the microbial communities was determined by the combined effects of several factors. Among all the environmental parameters, the pH had a significant influence on the microbial communities at the phylum, class and genus level (*P* < 0.05), which indicated that the pH would have a more powerful influence on highly abundant species^45^.

### Significant differences in microbial communities

Biomarker analysis was performed using LEfSe to determine the significant differences in microbial abundance between saltwater and freshwater. As depicted in Supplementary Fig. S3A, a total of 14 microbial clades had significant differences with an LDA threshold value of 4.0 (Supplementary Fig. S3B). Most of the microbes were significantly more abundant in freshwater samples, while only two clades were enriched in saltwater samples. Notably, *Alteromonadales* (order) and *Marinobacter* (genus) were enriched in the saline water samples (TDS > 10 g/L) (Supplementary Fig. S3C, P < 0.05), known as halophilic or halotolerant microorganisms^45^, which usually live in marine environments. The aquifer in the PRD has been confirmed to have a high salinity, and a previous ^14^C analysis concluded that the saltwater in the confined PRD aquifer most likely originated from seawater during the Holocene transgression period^26^. Many studies have demonstrated that the PRD underwent at least two large-scale transgressions during the Holocene period^22,26,28^, leading to a long period of interaction between the paleo-seawater and groundwater. Although some researchers concluded that the groundwater of the PRD has been undergoing freshening^22,26^ during deltaic evolution of the PRD, we found that the aquifers of the PRD still contained saline water on a large scale and contained some microbes that live in the marine environment, such as *Alteromonadales* (order) and *Marinobacter* (genus). Thus, it can be concluded that the groundwater environment of high salinity originated from seawater^26,27^, and this impact of the seawater intrusion has lasted to this day. In contrast, the freshwater samples in our study were primarily dominated by *Bacteroidetes* (phylum) and *β-proteobacteria* (class). In general, *β-proteobacteria* is a freshwater environmental indicator species, and many studies have documented that *β-proteobacteria* predominate in low salinity environments^46–48^. Simultaneously, it was again confirmed by LEfSe analysis that the abundance of *β-proteobacteria* was larger in the freshwater samples, which is consistent with the results of the microbial community composition analysis (Fig. 3B). In addition, there were three orders, three families and four genera that were enriched in the freshwater samples, indicating that most of the microbes were more likely to survive in a low salinity environment^49^.

### Microbial functional predictive analysis

**Figure 4:**
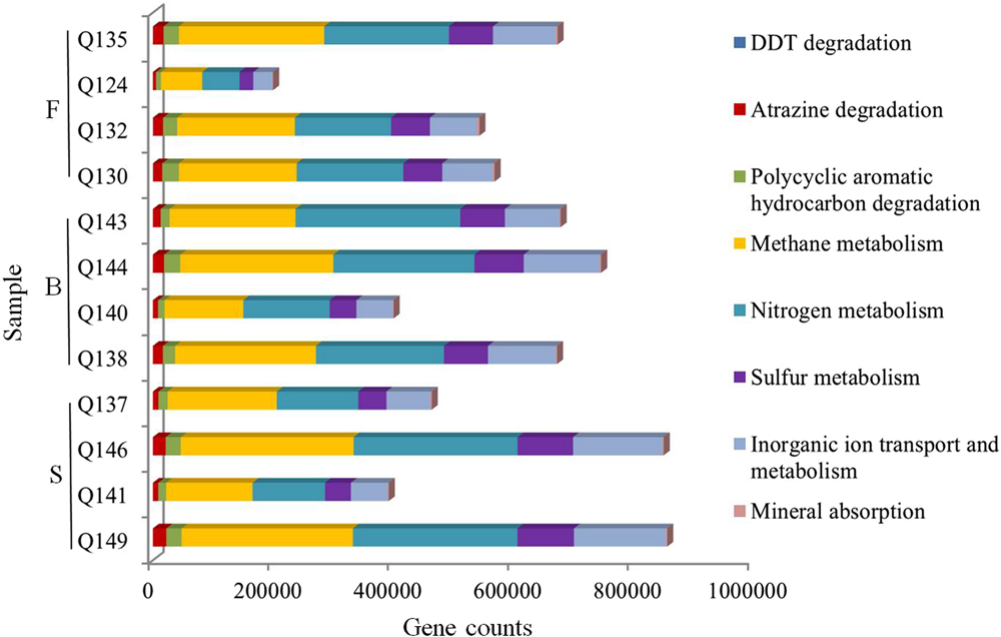
The relative abundance of some predicted functional profiles in the groundwater samples examined based on KEGG orthologous groups.

Based on the KEGG orthologous groups^50^, the functional profiles of the microbial communities were predicted but not measured for the 12 groundwater samples. At the metabolic pathway level, the primary functions included the degradation of pesticides and refractory organics (dichlorodiphenyltrichloroethane (DDT), atrazine and polycyclic aromatic hydrocarbons), matter cycling (methane, nitrogen and sulfur), as well as inorganic ion and mineral metabolism (Fig. 4). These underground microorganisms would have some level of capacity for natural pollutants degradation. In addition, this study also revealed that the microbes participated widely in various metabolic pathways, such as carbon, nitrogen and sulfur circulation, which might be attributed to the genera *Methanococcus*, *Candidatus_Nitrosoarchaeum* and *Desulfovibrio* (Fig. 3C) and had an important role in cycling constituents and the maintenance of the environmental balance. In addition, the phylum *Proteobacteria*, which was involved in nitrogen cycling^51^ was dominant in all the samples. Sulfate-reducing bacteria (SRB), which play an important role in the biodegradation of organic matter,^52^ have been detected in groundwater (Fig. 3C). Although microorganisms have some natural purification capacity, naturally occurring ammonia nitrogen has been estimated to be up to 8600 × 10^6^ kg in the aquitard of the PRD^27^. A substantial amount of ammonium was expected to be released from the aquitard^27^. The Guangdong Hydrogeology Team found that the groundwater in PRD contains ammonium at concentrations as high as 560 mg/L. The ammonium originated in the overlying organic-rich Holocene-Pleistocene aquitard and entered the aquifer through groundwater transport and diffusion^27^. Due to the low-permeability of the aquitard and the low rate of recharge^53^, the large amount of ammonium may be gradually migrating into the river water and coastal seawater, which may disrupt the ecological balance and cause substantial harm to the environment.

## Conclusion

In this study, we integrated methodologies from hydrogeology, ecology and microbiology and applied them to study the physicochemical water parameters and microbial changes induced by saltwater intrusion in groundwater. Most previous studies on microbial ecology across salinity gradients or in hypersaline environments focused on salt lakes, wetlands and marine environments. Only a few studies addressed the diversity of the structure, the distribution of the microbial species, and their relationship based on different salt concentrations with a large spatial scale in groundwater environment. Our study demonstrated that salinity was the primary driving force of the microbial community composition in the groundwater, but the alpha diversity did not completely follow the salinity gradient, and there were many other co-varying factors that could also influence the formations of bacterial and archaeal communities, such as the pH and TDS, as well as the TN. The most abundant phylum in the groundwater of the PRD area was *Proteobacteria*, followed by *Firmicutes* and *Bacteroidetes*, and the community structure of the archaea was dominated by *Thaumarchaeota* and *Euryarchaeota* which were more enriched in some saline water.

Our results indicated that the microbial community composition varied significantly along the aquifer salinity. The genera *Acidovorax, Aquabacterium, Denitratisoma* and *Comamonas* had a relatively high abundance in freshwater samples, while the saltwater samples contained more genera related to carbon, nitrogen and sulfur cycles, such as *Methanococcus*, *Candidatus_Nitrosoarchaeum* and *Desulfovibrio*. The results of the high-throughput sequencing and the functional genes (*mcrA* and *dsrA*) illustrated that sulfate reducers and methanogens were present in the groundwater, which may be responsible for the gas generation of H_2_S and CH_4_ in the stratum. Although some researchers concluded that the groundwater of the PRD has been undergoing freshening during deltaic evolution of the PRD, we found that the aquifers of the PRD still contained large amounts of saltwater and contained some microbes that live in marine environments, such as *Alteromonadales* (order) and *Marinobacter* (genus), indicating that the impact of the seawater intrusion has lasted to this day.

Additionally, the predictive metagenomic analysis showed that the metabolic pathway included the degradation of pesticides and refractory organics (DDT, atrazine and polycyclic aromatic hydrocarbons), matter cycling (methane, nitrogen and sulfur), as well as inorganic ion and mineral metabolism that were present in the PRD aquifer. To our knowledge, this may be the first report about the microbial communities in groundwater along a salinity gradient in the PRD area using a high-throughput sequencing approach. Therefore, this study provides a baseline measurement of the prokaryotic microbial community in groundwater affected by seawater intrusion, laying a foundation for further study on the ecological characteristics of microorganism along a salinity gradient in the aquifer.

## Methods

### Site description, sample collection and physicochemical analysis

**Figure 5:**
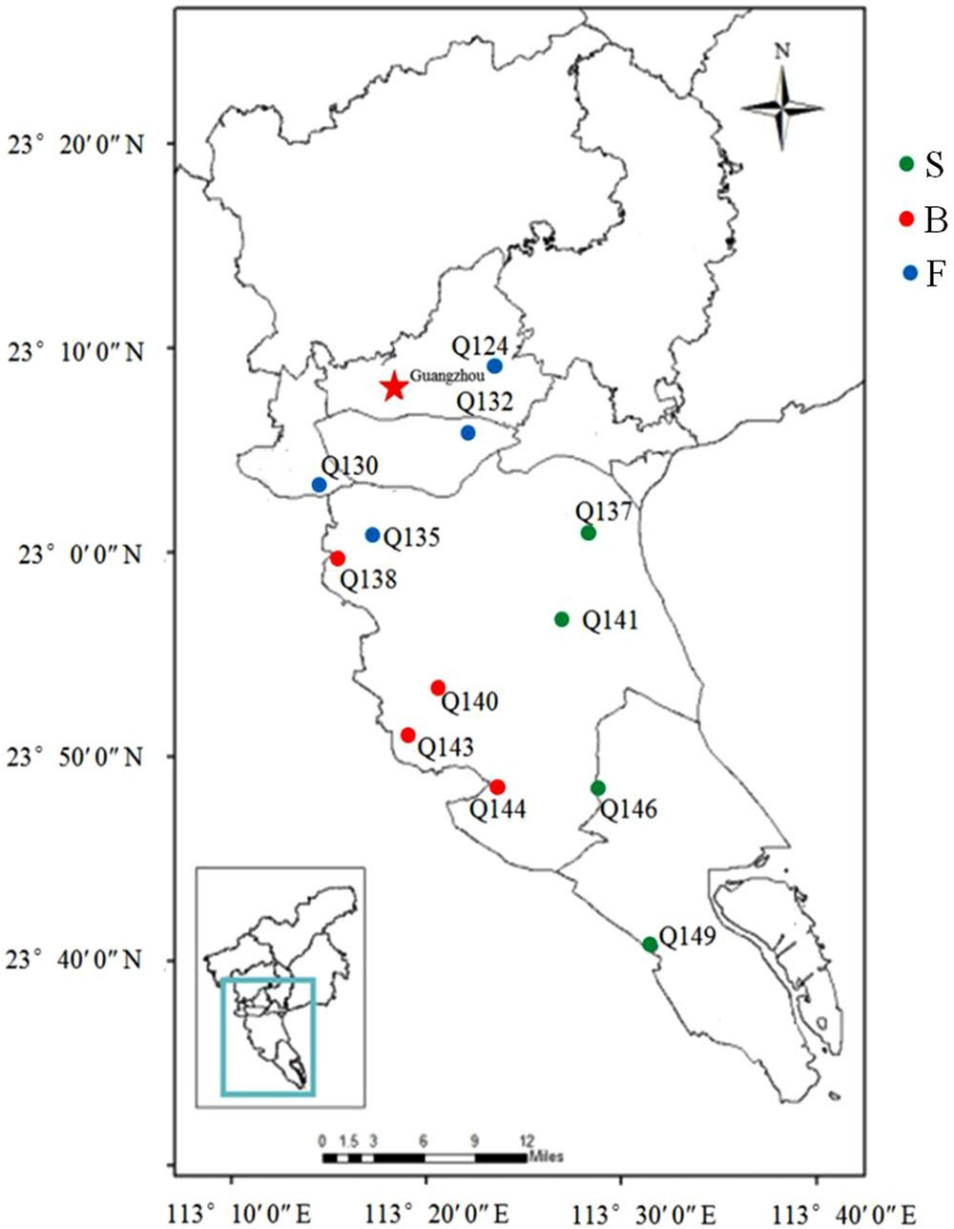
Map showing the sampling stations of all 12 groundwater samples. (freshwater (F: TDS < 1 g/L); brackish water (B: 1 g/L < TDS < 10 g/L); saline water (S: TDS > 10 g/L).

Twelve sampling sites were selected to collect samples on May 10, 2017 in the central and southern regions of the PRD area. The twelve sampling sites include four saline monitoring wells (S) with TDS > 10 g/L, four brackish monitoring wells (B) with 1 g/L < TDS < 10 g/L, and four freshwater monitoring wells (F) with TDS < 1 g/L as shown in Fig. 5. The PRD has a subtropical monsoon climate, which is warm and humid all year round with an annual average temperature of 22 °C. The annual average precipitation ranges from 1,600 to 2,000 mm, primarily from April to October. According to the hydrogeologic survey of the PRD provided by the Guangdong Geological Survey^42^, there is a deposition consist of very fine-grained silt and clay overlying the terrestrial aquifer^28^. The thickness of the deposition units was between 5 and 20 m. All the collected groundwater samples were below 10 m overlying a thickness of deposition units. Hence, the water type of groundwater samples collected were confined aquifer. The investigated lithostratigraphic groups all belonged to the quaternary. The detailed information for each well is shown in Supplementary Table S6.

The wells were cleaned by pumping groundwater (three times well volumes) with an electric submersible pump to remove stagnant water before sampling. The physicochemical parameters, such as oxygen content (DO), oxidation-reduction potential (ORP), TDS, electrical conductivity (EC), pH and temperature (T) were measured using the freshly collected groundwater samples using a portable meter (Hanna Instrument, Milan, Italy). All the groundwater samples were collected in sterile 10-L plastic containers for filtration to collect the microbial samples, and an additional 500 mL for each sample was collected in triplicate for physicochemical analyses. All samples were kept at 4 °C during transportation and were refrigerated until they were used. Water samples used for the physicochemical analyses were filtered through a sterile 0.45-μm nitrocellulose membrane filter (Millipore, Sigma., Burlington, MA, USA) using a vacuum system. Physicochemical analysis was performed according to international standards^54^. Cations (K^+^, Ca^2+^, Na^+^ and Mg^2+^) were measured using Inductively Coupled Plasma Optical Emission Spectrometry (ICAP 7600, ICP-OES; Thermo Fisher Scientific, Waltham, MA, USA). Anions (NO_3_^−^, Cl^−^ and SO_4_^2−^) were measured using ion chromatography (Swiss Wantong type 883 chromatograph; Metrohm Schweiz AG, Zofingen, Switzerland). HCO_3_^−^ was measured using acid-based titration analysis (DZ/T 0064.49–93). Total nitrogen (TN) was detected using the alkaline potassium persulfate digestion and UV spectrophotometric method; total phosphorus (TP) was detected using the persulfate digestion and spectrophotometric method; total organic carbon (TOC) was measured using a total carbon analyzer (Elementar, Liquid TOCII; Elementar Analysensysteme GmbH, Langenselbold, Germany)^55^.

### DNA extraction and PCR amplification

Total DNA was extracted from 5 L of water filtered through a sterile 0.2-μm nitrocellulose membrane filter (Millipore, Sigma., Burlington, MA, USA) using a vacuum system. A MOBIO PowerSoil® DNA Isolation Kit (Qiagen/MO BIO Laboratories Inc., Carlsbad, CA, USA) was used to extract the DNA. The V4 region of the prokaryotic microbial 16S rRNA gene was amplified by PCR using the forward primer 515F (5′-GTGCCAGCMGCCGCGGTAA-3′) and the reverse primer 806R (5′-GGACTACHVGGGTWTCTAAT-3′)^56^. The functional gene *mcrA* of the methanogens was amplified by PCR using the forward primer ME1-F (5′- GCMATGCARATHGGWATGTC-3′) and the reverse primer ME2-R (5′-TCATKGCRTAGTTDGGRTA-3′)^57^. The functional gene *dsrA* of the sulfate reducers was amplified by PCR using the forward primer dsrA 290-F (5′-CGGCGTTGCGCATTTYCAYACVVT-3′) and the reverse primer dsrA 660-R (5′-GCCGGACGATGCAGHTCRTCCTGRWA-3′)^58^. PCR reactions were conducted on a BioRad S1000 thermocycler (Bio-Rad Laboratories Inc., Hercules, CA, USA) under the following conditions: 94 °C for 5 min; 30 cycles: 94 °C for 30 s, 52 °C for 30 s and 72 °C for 30 s; and 72 °C for 10 min. Amplicons were extracted from 1.0% agarose gels and purified using an EZNA Gel Extraction Kit (Omega, Bio-Tek, Norcross, GA, USA) according to the manufacturer’s instructions. Libraries were prepared using an NEBNext® Ultra™ DNA Library Prep Kit for Illumina® (New England Biolabs, Ipswich, MA, USA) according to the manufacturer’s instructions, and sequencing was performed on an Illumina HiSeq 2500 system at Magi Gene Technology (Guangzhou, China).

### Sequence processing and statistical analysis

Paired-end raw reads were demultiplexed, quality-filtered by the Trimmomatic software and merged by the Fast Length Adjustment of SHort reads (FLASH) software using the following criteria: (i) The reads were truncated at any site receiving an average quality score <20 over a 50 bp sliding window; (ii) The primers were exactly matched allowing 2 nucleotide mismatching, and reads containing ambiguous bases were removed, and (iii) The sequences that overlap longer than 10 bp were merged according to their overlap sequence. The quality sequences were assigned into operational taxonomic units (OTUs) with a 97% similarity cutoff in the UPARSE platform^59^, and chimeric microbial sequences were screened using UCHIME^60^. The taxonomy of each 16S rRNA gene sequence was analyzed by the RDP Classifier algorithm (http://rdp.cme.msu.edu/) against the Silva 16S rRNA database using a confidence threshold of 70%^61^.

Rarefaction curves were plotted for each sample based on the OTU information^62^. Alpha-diversity analyses, including community diversity indices (Shannon and Simpson), community richness parameters (Chao and ACE), community evenness indices (Heip), as well as a sequencing depth index (Good’s coverage), were performed using Mothur software^63^. In addition, the beta-diversity of the groundwater samples was determined based on the unweighted-unifrac distance including principal co-ordinate analysis (PCoA) and hierarchical clustering analysis using the Quantitative Insights Into Microbial Ecology (Qiime 1.7.0) software^64^. The associations between physicochemical variables and microbial community structure were determined by Spearman’s rank correlation analysis using the SPSS software (IBM Corp., Armonk, NY, USA), and the corresponding heatmap was obtained using the plots package in R^65^. Redundancy analysis (RDA) was performed to reveal microbe-environment relations with the CANOCO 4.5 software (Biometris, Wageningen, The Netherlands). *P*-values < 0.05 were considered statistically significant.

The potential biomarkers analysis was performed by the linear discriminant analysis (LDA) effect size (LEfSe)^66^ using the Kruskal-Wallis test to determine the significant differences between the saltwater and freshwater. LDA was performed to assess the difference of each microbial taxon with an LDA threshold value of 4.0. In addition, the OTU sequences were normalized by phylogenetic investigation of the communities by the reconstruction of unobserved states (PICRUSt) and compared to the KEGG databases for functional predictive analysis^50^.

### Data Access

All the raw sequence data were deposited in the NCBI Sequence Read Archive (SRA) under the accession number SRP118856.

## Electronic supplementary material

Supplementary materials

## Data Availability

The data analyzed during this study are included in this published article (and its Supplementary Information files).
