# Supplementary material for: Diversity and predictive metabolic pathways of the prokaryotic microbial community along a groundwater salinity gradient of the Pearl River Delta, China
Source: Sci Rep. 2018 Nov 23;8:17317. doi: 10.1038/s41598-018-35350-2 (PMC6251883; doi:10.1038/s41598-018-35350-2)
Supplement: Supplementary file 1 — Supplementary materials [file 41598_2018_35350_MOESM1_ESM.docx]

**Diversity and predictive metabolic pathways of** **the prokaryotic microbial community along a groundwater salinity gradient of the Pearl River Delta, China**

**Shilei Sang****^1^, Xiaoying Zhang^1^, Heng Dai^2^****^*^, Bill X.Hu^2*^, Hao Ou^1^, Liwei Sun^1^**

^*^corresponding author: [heng.dai@jnu.edu.cn](mailto:heng.dai@jnu.edu.cn), Bill.x.hu@gmail.com

^1^Department of Ecology, Jinan University, Guangzhou 510632, Guangdong, China

^2^Institute of Groundwater and Earth Sciences, Jinan University, Guangzhou 510632, Guangdong, China


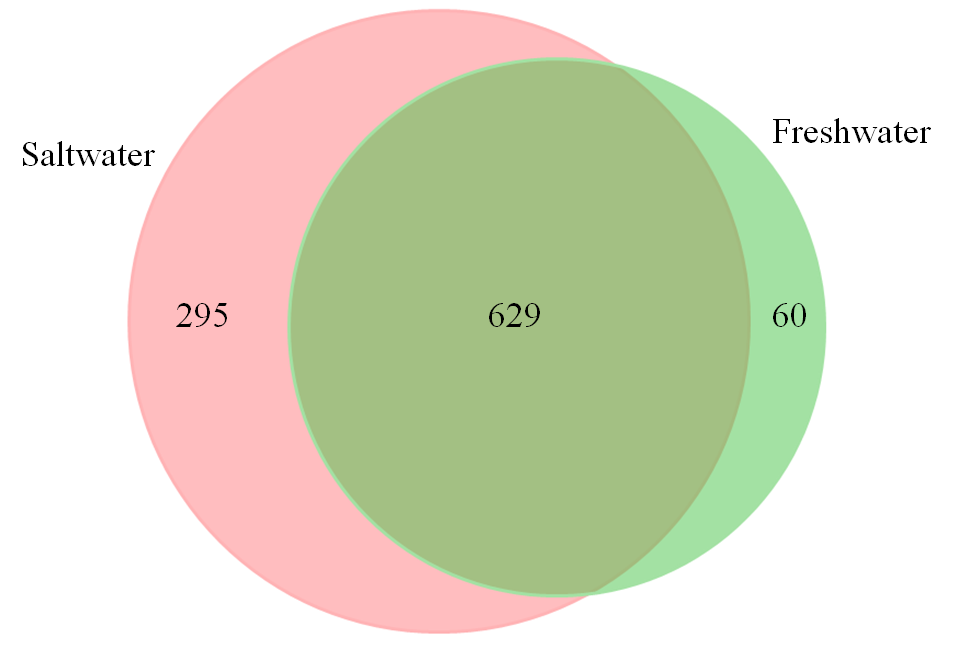


**Supplementary Fig. S1** Venn diagram exhibited the shared and unique OTUs between the saltwater and freshwater samples.


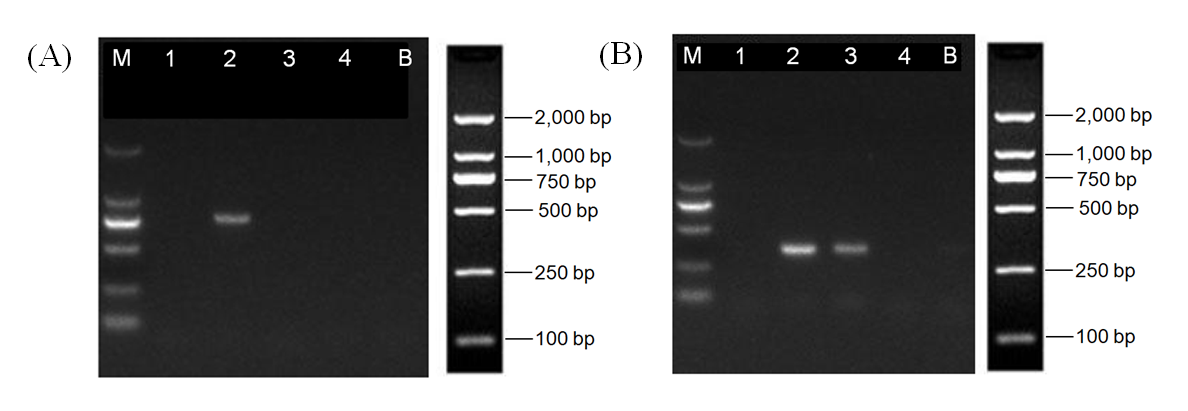


**Supplementary Fig. S2** (A) Detection of the functional gene (*mcrA*) by PCR of MA with a fragment size of 800 bp;

(B) Detection of the functional gene (*dsrA*) by PCR of SRB with a fragment size of 370 bp.

(M: Mark, DL2000, positive control; B: Blank control; 1-Q146, 2-Q137, 3-Q141, 4-Q149)

**
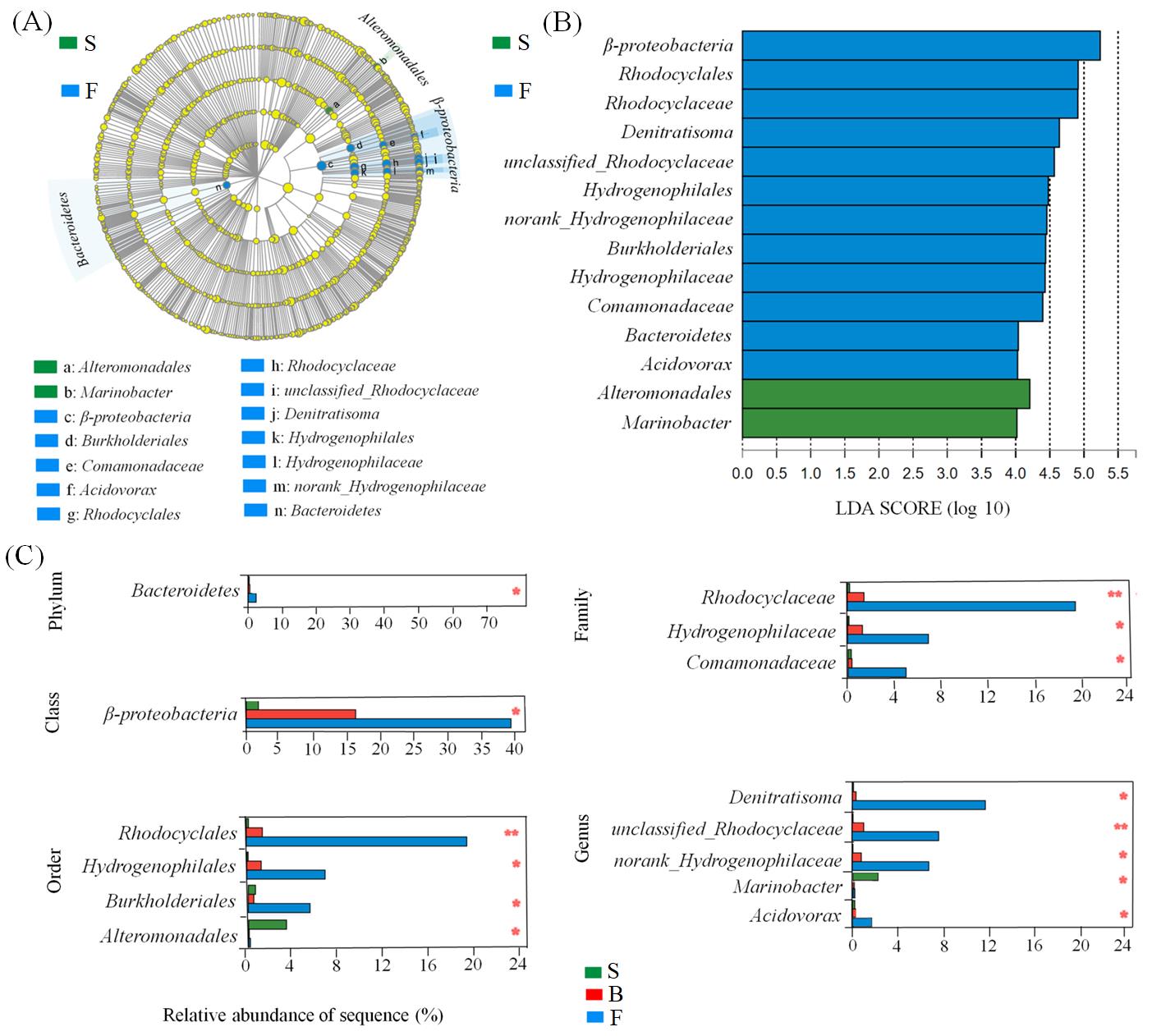
**

**Supplementary Fig. S3** Assessment of the difference in microbial abundance between the saltwater and freshwater samples using the LEfSe method. (A) The cladogram of the microbial taxa, from the phylum down to the genus levels. Green indicates the taxa enriched in saline water samples (S), and blue indicates the taxa enriched in fresh water samples (F). Brackish water samples (B) have no microbiota taxa identified; (B) The effect on the size of the difference was calculated by the linear discriminant analysis between saltwater and freshwater samples with a threshold value of 4.0. Green indicates saline water samples (S), and blue indicates fresh water samples (F); (C) The Kruskal-Wallis Test was used to calculate the microbial abundance with significant differences at the phylum, class, order, family, and genus level. Green indicates the taxa enriched in the saline water samples (S); red indicates the taxa enriched in the brackish water samples (B) and blue indicates the taxa enriched in fresh water samples (F) (*P＜0.05, **P＜0.01).

**Supplementary Table S1.** Alpha-diversity indices of all the 12 groundwater samples

| Sample | Reads | Label: 0.97 | | | | | | |
| --- | --- | --- | --- | --- | --- | --- | --- | --- |
|  |  | OTUs | Shannon | Simpson | Ace | Chao | Heip | Coverage |
| Q149 | 29355 | 379 | 2.262574 | 0.202238 | 516.075362 | 496.924051 | 0.022772 | 0.993544 |
| Q141 | 29355 | 634 | 3.879883 | 0.09219 | 756.776935 | 785 | 0.074911 | 0.992884 |
| Q137 | 29355 | 556 | 3.772044 | 0.062227 | 678.176183 | 656.714286 | 0.07652 | 0.993356 |
| Q146 | 29355 | 365 | 1.971665 | 0.252025 | 516.052446 | 496.486486 | 0.016985 | 0.993403 |
| Q138 | 29355 | 488 | 2.902548 | 0.140526 | 647.599968 | 647 | 0.03536 | 0.992507 |
| Q140 | 29355 | 364 | 2.664574 | 0.128818 | 661.014276 | 606.25 | 0.036809 | 0.99279 |
| Q144 | 29355 | 466 | 2.725767 | 0.172811 | 597.322945 | 613 | 0.030684 | 0.993073 |
| Q143 | 29355 | 410 | 2.583783 | 0.231144 | 535.456066 | 522.039474 | 0.029944 | 0.993827 |
| Q130 | 29355 | 429 | 3.396037 | 0.075319 | 575.16672 | 586.229508 | 0.067396 | 0.99345 |
| Q132 | 29355 | 462 | 3.667041 | 0.055142 | 575.001869 | 552.517241 | 0.082724 | 0.994062 |
| Q124 | 29355 | 379 | 2.392549 | 0.215975 | 558.995606 | 530.214286 | 0.0263 | 0.99312 |
| Q135 | 29355 | 474 | 3.629614 | 0.074655 | 581.905527 | 564.012195 | 0.077586 | 0.994251 |

**Supplementary Table S2.** The mean differences between the groups were compared by independent t-test (α=0.05) using SPSS 19.0. There are not significant differences in all kinds of alpha-diversity indexes (P＞0.05).

| Estimators | S-Mean | S-Sd | B-Mean | B-Sd | F-Mean | F-Sd | Pvalue (S-B) | Qvalue (S-B) | Pvalue (F-B) | Qvalue (F-B) | Pvalue (F-S) | Qvalue (F-S) |
| --- | --- | --- | --- | --- | --- | --- | --- | --- | --- | --- | --- | --- |
| OTUs | 483.5 | 132.75 | 432 | 55.976 | 436 | 42.497 | 0.5015 | 0.9264 | 0.9131 | 0.9131 | 0.5209 | 0.6078 |
| Shannon | 2.9715 | 0.9947 | 2.7192 | 0.1354 | 3.2713 | 0.5980 | 0.6330 | 0.9264 | 0.1218 | 0.2813 | 0.6239 | 0.6239 |
| Simpson | 0.1522 | 0.0898 | 0.1683 | 0.0458 | 0.1053 | 0.0744 | 0.7594 | 0.9264 | 0.1991 | 0.2813 | 0.4517 | 0.6078 |
| Ace | 616.77 | 120.63 | 610.35 | 56.96 | 572.77 | 9.7283 | 0.9264 | 0.9264 | 0.2411 | 0.2813 | 0.4945 | 0.6078 |
| Chao | 608.78 | 139.61 | 597.07 | 53.106 | 558.24 | 23.345 | 0.8806 | 0.9264 | 0.2292 | 0.2813 | 0.502 | 0.6078 |
| Heip | 0.0478 | 0.0323 | 0.0332 | 0.0034 | 0.0635 | 0.0256 | 0.4037 | 0.9264 | 0.0574 | 0.2813 | 0.4752 | 0.6078 |
| Coverage | 0.9933 | 0.0003 | 0.9931 | 0.0006 | 0.9937 | 0.0005 | 0.4659 | 0.9264 | 0.1335 | 0.2813 | 0.2069 | 0.6078 |

**Supplementary Table S3.** The relative abundances of dominant phyla (abundance > 2% at least one sample) in all samples.

| Phylum taxon | Q149 | Q141 | Q137 | Q146 | Q138 | Q140 | Q144 | Q143 | Q130 | Q132 | Q124 | Q135 |
| --- | --- | --- | --- | --- | --- | --- | --- | --- | --- | --- | --- | --- |
| *Bacteroidetes* | 0.14% | 0.33% | 0.38% | 0.20% | 0.36% | 0.17% | 0.94% | 0.35% | 0.95% | 5.69% | 1.73% | 0.48% |
| *Thaumarchaeota* | 0.12% | 29.02% | 0.46% | 0.17% | 10.13% | 0.09% | 0.13% | 0.09% | 0.19% | 0.19% | 0.04% | 0.14% |
| *Firmicutes* | 37.40% | 9.79% | 10.38% | 42.52% | 31.35% | 18.77% | 35.89% | 12.17% | 10.42% | 7.16% | 7.60% | 23.57% |
| *Nitrospirae* | 0.11% | 0.67% | 0.59% | 0.09% | 2.05% | 0.06% | 0.09% | 0.09% | 0.11% | 0.08% | 0.08% | 3.28% |
| *Euryarchaeota* | 0.03% | 0.04% | 13.09% | 0.01% | 0.00% | 0.00% | 0.10% | 0.01% | 0.21% | 0.00% | 0.01% | 0.05% |
| *Proteobacteria* | 61.18% | 50.34% | 65.94% | 56.15% | 53.06% | 80.11% | 60.07% | 85.50% | 86.51% | 84.13% | 85.40% | 64.78% |
| *Woesearchaeota* | 0.00% | 2.05% | 0.06% | 0.00% | 0.16% | 0.00% | 0.00% | 0.00% | 0.00% | 0.00% | 0.00% | 0.00% |
| unclassified*_Bacteria* | 0.04% | 0.53% | 0.21% | 0.02% | 0.14% | 0.02% | 0.19% | 0.03% | 0.03% | 0.04% | 3.55% | 0.88% |
| other phyla | 0.98% | 7.23% | 8.89% | 0.84% | 2.75% | 0.78% | 2.59% | 1.76% | 1.58% | 2.71% | 1.59% | 6.82% |

**Supplementary Table S4.** The relative abundances of the 12 presentative classes (abundance > 2% at least one sample) in all 12 samples.

| Class taxon | Q149 | Q141 | Q146 | Q137 | Q138 | Q140 | Q144 | Q143 | Q130 | Q132 | Q124 | Q135 |
| --- | --- | --- | --- | --- | --- | --- | --- | --- | --- | --- | --- | --- |
| *α-proteobacteria* | 2.06% | 9.88% | 1.44% | 3.67% | 2.73% | 0.99% | 11.67% | 3.35% | 4.68% | 6.89% | 1.22% | 2.71% |
| *Bacilli* | 37.39% | 9.65% | 42.51% | 9.68% | 31.29% | 18.75% | 35.84% | 12.16% | 10.41% | 7.14% | 7.59% | 22.16% |
| *β-proteobacteria* | 1.24% | 2.59% | 1.26% | 2.12% | 8.41% | 43.68% | 4.18% | 9.81% | 39.26% | 29.76% | 72.29% | 18.45% |
| *δ-proteobacteria* | 0.57% | 6.02% | 0.42% | 34.22% | 2.14% | 1.94% | 0.88% | 0.78% | 0.40% | 0.38% | 0.51% | 8.77% |
| *ε-proteobacteria* | 0.19% | 0.61% | 0.14% | 0.79% | 0.17% | 13.50% | 0.13% | 46.63% | 0.12% | 1.47% | 0.09% | 0.16% |
| *γ-proteobacteria* | 57.11% | 30.57% | 52.87% | 25.09% | 39.52% | 19.90% | 43.19% | 24.90% | 41.89% | 45.52% | 11.01% | 34.65% |
| *Marine Group I* | 0.02% | 27.44% | 0.02% | 0.02% | 9.35% | 0.00% | 0.02% | 0.01% | 0.02% | 0.01% | 0.01% | 0.03% |
| *Methanococci* | 0.00% | 0.01% | 0.00% | 12.47% | 0.00% | 0.00% | 0.00% | 0.00% | 0.00% | 0.00% | 0.00% | 0.00% |
| *Nitrospira* | 0.11% | 0.67% | 0.09% | 0.59% | 2.05% | 0.06% | 0.09% | 0.09% | 0.11% | 0.08% | 0.08% | 3.28% |
| *norank_Woesearchaeota* | 0.00% | 2.05% | 0.00% | 0.06% | 0.16% | 0.00% | 0.00% | 0.00% | 0.00% | 0.00% | 0.00% | 0.00% |
| *Sphingobacteriia* | 0.12% | 0.28% | 0.15% | 0.19% | 0.20% | 0.16% | 0.89% | 0.25% | 0.93% | 5.48% | 1.69% | 0.13% |
| *unclassified_Bacteria* | 0.04% | 0.53% | 0.02% | 0.21% | 0.14% | 0.02% | 0.19% | 0.03% | 0.03% | 0.04% | 3.55% | 0.88% |
| other classes | 1.15% | 9.69% | 1.08% | 10.89% | 3.85% | 0.98% | 2.91% | 1.98% | 2.13% | 3.23% | 1.97% | 8.78% |

**Supplementary Table S5.** The relative abundances of the top 50 genera in all 12 samples

| Genus taxon | Q149 | Q141 | Q137 | Q146 | Q138 | Q140 | Q144 | Q143 | Q130 | Q132 | Q124 | Q135 |
| --- | --- | --- | --- | --- | --- | --- | --- | --- | --- | --- | --- | --- |
| *Exiguobacterium* | 36.13% | 9.18% | 9.24% | 40.96% | 30.15% | 18.22% | 34.13% | 11.78% | 9.96% | 6.60% | 7.04% | 21.17% |
| *Acinetobacter* | 16.09% | 7.65% | 7.67% | 19.16% | 12.82% | 6.88% | 17.51% | 5.43% | 8.99% | 19.12% | 5.00% | 12.03% |
| *unclassified_Enterobacteriaceae* | 20.67% | 5.56% | 4.77% | 22.63% | 15.74% | 6.69% | 17.00% | 6.28% | 5.30% | 4.06% | 3.32% | 10.46% |
| *Pseudomonas* | 10.18% | 7.48% | 8.25% | 9.84% | 7.77% | 5.36% | 7.33% | 6.69% | 2.56% | 2.01% | 1.91% | 10.12% |
| *Sulfuricurvum* | 0.16% | 0.12% | 0.49% | 0.13% | 0.15% | 12.31% | 0.11% | 46.35% | 0.11% | 0.44% | 0.08% | 0.13% |
| *unclassified_Gallionellaceae* | 0.26% | 0.22% | 0.55% | 0.21% | 4.12% | 39.76% | 0.25% | 2.25% | 7.81% | 0.65% | 0.93% | 0.43% |
| *Denitratisoma* | 0.06% | 0.05% | 0.06% | 0.06% | 0.09% | 0.09% | 0.36% | 0.06% | 0.81% | 0.16% | 39.63% | 0.65% |
| *unclassified_Rhodocyclaceae* | 0.08% | 0.08% | 0.10% | 0.10% | 0.22% | 0.41% | 2.42% | 1.48% | 13.93% | 5.09% | 7.38% | 10.14% |
| *Aeromonas* | 0.19% | 0.16% | 0.09% | 0.33% | 0.23% | 0.18% | 0.26% | 2.64% | 19.98% | 16.82% | 0.13% | 0.20% |
| *norank_Hydrogenophilaceae* | 0.04% | 0.06% | 0.08% | 0.04% | 0.05% | 0.13% | 0.08% | 2.84% | 0.51% | 0.16% | 21.75% | 4.08% |
| *Candidatus*_*Nitrosoarchaeum* | 0.01% | 27.21% | 0.01% | 0.01% | 0.22% | 0.00% | 0.01% | 0.01% | 0.01% | 0.00% | 0.01% | 0.01% |
| *Desulfovibrio* | 0.21% | 0.08% | 15.44% | 0.04% | 0.07% | 0.17% | 0.26% | 0.06% | 0.03% | 0.06% | 0.13% | 3.11% |
| *Methanococcus* | 0.00% | 0.01% | 12.47% | 0.00% | 0.00% | 0.00% | 0.00% | 0.00% | 0.00% | 0.00% | 0.00% | 0.00% |
| *norank*_*Methylophilaceae* | 0.02% | 0.05% | 0.01% | 0.04% | 0.07% | 0.04% | 0.02% | 0.02% | 1.17% | 8.64% | 0.04% | 0.04% |
| *norank_Marine Group I* | 0.01% | 0.23% | 0.01% | 0.00% | 9.13% | 0.00% | 0.01% | 0.00% | 0.01% | 0.00% | 0.00% | 0.01% |
| *unclassified*_*Desulfobulbaceae* | 0.03% | 1.93% | 6.32% | 0.01% | 0.13% | 0.02% | 0.25% | 0.01% | 0.02% | 0.01% | 0.01% | 0.15% |
| *Marinobacter* | 8.21% | 0.16% | 0.06% | 0.04% | 0.02% | 0.03% | 0.03% | 0.02% | 0.03% | 0.02% | 0.00% | 0.05% |
| *unclassified*_*Betaproteobacteria* | 0.19% | 0.09% | 0.15% | 0.18% | 0.64% | 1.31% | 0.14% | 0.42% | 2.78% | 2.05% | 0.19% | 0.19% |
| *Sphingobium* | 0.08% | 4.91% | 1.44% | 0.09% | 0.05% | 0.05% | 0.11% | 0.08% | 0.08% | 0.07% | 0.04% | 1.30% |
| *Acidovorax* | 0.03% | 0.04% | 0.03% | 0.05% | 0.06% | 0.11% | 0.03% | 0.05% | 6.61% | 0.82% | 0.12% | 0.18% |
| *Comamonas* | 0.03% | 0.08% | 0.03% | 0.03% | 0.07% | 0.06% | 0.06% | 0.07% | 2.58% | 4.73% | 0.10% | 0.12% |
| *Sediminibacterium* | 0.02% | 0.02% | 0.04% | 0.01% | 0.02% | 0.02% | 0.68% | 0.04% | 0.24% | 4.81% | 1.50% | 0.00% |
| *Nitrospira* | 0.11% | 0.67% | 0.59% | 0.09% | 2.05% | 0.06% | 0.09% | 0.09% | 0.11% | 0.08% | 0.08% | 3.28% |
| *Desulfonatronum* | 0.13% | 0.01% | 5.89% | 0.06% | 0.00% | 0.02% | 0.02% | 0.00% | 0.01% | 0.02% | 0.01% | 0.02% |
| *Bacillus* | 0.82% | 0.29% | 0.26% | 0.98% | 0.73% | 0.33% | 0.85% | 0.24% | 0.25% | 0.22% | 0.22% | 0.61% |
| *Methylomonas* | 0.01% | 0.06% | 0.04% | 0.01% | 0.04% | 0.01% | 0.04% | 0.03% | 4.05% | 1.43% | 0.02% | 0.04% |
| *unclassified*_*Bacteria* | 0.04% | 0.53% | 0.21% | 0.02% | 0.14% | 0.02% | 0.19% | 0.03% | 0.03% | 0.04% | 3.55% | 0.88% |
| *Methylocystis* | 0.02% | 0.05% | 0.05% | 0.02% | 0.08% | 0.01% | 1.23% | 0.02% | 0.63% | 3.39% | 0.02% | 0.05% |
| *Shewanella* | 0.05% | 4.01% | 0.67% | 0.04% | 0.02% | 0.02% | 0.02% | 0.00% | 0.02% | 0.05% | 0.01% | 0.43% |
| *Sphingomonas* | 0.35% | 0.90% | 0.51% | 0.33% | 0.45% | 0.25% | 0.53% | 0.35% | 0.41% | 0.65% | 0.21% | 0.42% |
| *unclassified*_*Methylophilaceae* | 0.07% | 0.10% | 0.08% | 0.03% | 0.07% | 0.22% | 0.05% | 0.04% | 0.29% | 3.22% | 0.09% | 0.13% |
| *Magnetovibrio* | 0.03% | 0.82% | 0.05% | 0.00% | 0.01% | 0.26% | 2.69% | 0.01% | 0.02% | 0.02% | 0.00% | 0.02% |
| *Syntrophus* | 0.01% | 0.06% | 0.01% | 0.00% | 0.26% | 0.01% | 0.03% | 0.01% | 0.01% | 0.01% | 0.01% | 3.49% |
| *norank*_*Soil* *Crenarchaeotic* *Group* | 0.10% | 1.44% | 0.41% | 0.14% | 0.73% | 0.08% | 0.10% | 0.08% | 0.16% | 0.18% | 0.03% | 0.11% |
| *Geobacter* | 0.05% | 0.11% | 0.41% | 0.03% | 0.07% | 1.55% | 0.07% | 0.05% | 0.12% | 0.07% | 0.03% | 0.43% |
| *unclassified*_*Gammaproteobacteria* | 0.05% | 1.98% | 0.07% | 0.02% | 0.08% | 0.05% | 0.06% | 0.30% | 0.11% | 0.05% | 0.00% | 0.19% |
| *Thiobacillus* | 0.01% | 0.07% | 0.08% | 0.08% | 0.03% | 0.08% | 0.08% | 1.66% | 0.13% | 0.08% | 0.13% | 0.21% |
| *Novosphingobium* | 0.02% | 0.22% | 0.04% | 0.01% | 0.65% | 0.00% | 0.95% | 0.49% | 0.02% | 0.14% | 0.02% | 0.05% |
| *norank*_*Gallionellaceae* | 0.01% | 0.03% | 0.02% | 0.00% | 1.80% | 0.41% | 0.00% | 0.03% | 0.19% | 0.04% | 0.02% | 0.01% |
| *norank*_*Nitrosomonadaceae* | 0.11% | 0.19% | 0.10% | 0.15% | 0.20% | 0.27% | 0.16% | 0.23% | 0.28% | 0.36% | 0.23% | 0.24% |
| *norank*_*Woesearchaeota* | 0.00% | 2.05% | 0.06% | 0.00% | 0.16% | 0.00% | 0.00% | 0.00% | 0.00% | 0.00% | 0.00% | 0.00% |
| *norank*_*Anaerolineaceae* | 0.06% | 0.46% | 0.51% | 0.04% | 0.19% | 0.06% | 0.08% | 0.49% | 0.04% | 0.10% | 0.08% | 0.08% |
| *Aquabacterium* | 0.01% | 0.07% | 0.02% | 0.02% | 0.01% | 0.01% | 0.03% | 0.03% | 0.17% | 1.72% | 0.02% | 0.04% |
| *unclassified*_*Rhodospirillaceae* | 0.01% | 0.11% | 0.01% | 0.00% | 0.01% | 0.00% | 1.62% | 0.00% | 0.08% | 0.05% | 0.24% | 0.00% |
| *unclassified*_*Rhodobacteraceae* | 0.92% | 0.17% | 0.08% | 0.07% | 0.05% | 0.02% | 0.08% | 0.63% | 0.02% | 0.02% | 0.01% | 0.06% |
| *Thiovirga* | 0.01% | 0.00% | 0.01% | 0.00% | 0.00% | 0.02% | 0.02% | 2.02% | 0.00% | 0.00% | 0.00% | 0.00% |
| *Vibrio* | 0.95% | 0.51% | 0.32% | 0.12% | 0.02% | 0.00% | 0.01% | 0.02% | 0.04% | 0.02% | 0.01% | 0.04% |
| *unclassified*_*Nitrospinaceae* | 0.01% | 1.62% | 0.00% | 0.00% | 0.41% | 0.00% | 0.00% | 0.00% | 0.00% | 0.01% | 0.00% | 0.00% |
| *Methylobacter* | 0.00% | 0.01% | 0.00% | 0.00% | 1.98% | 0.00% | 0.01% | 0.00% | 0.00% | 0.00% | 0.02% | 0.00% |
| *Enterococcus* | 0.28% | 0.08% | 0.08% | 0.36% | 0.28% | 0.12% | 0.31% | 0.09% | 0.10% | 0.05% | 0.04% | 0.23% |

**Supplementary Table S6.** The information of each well including station, depth of chimney filter and water type

| Well | Coordinates | | Depth of screened interval in well (m) | Type of aquifer |
| --- | --- | --- | --- | --- |
|  | E | N |  |  |
| Q149 | 113.53 | 22.68 | 28-52 | Confined |
| Q141 | 113.45 | 22.94 | 19.6-22.6 | Confined |
| Q137 | 113.47 | 23.02 | 13.8-34.2 | Confined |
| Q146 | 113.48 | 22.81 | 10.8-13.5 | Confined |
| Q138 | 113.26 | 22.99 | 19.8-21.9 | Confined |
| Q140 | 113.34 | 22.91 | 28-38.6 | Confined |
| Q144 | 113.39 | 22.81 | 36.5-55 | Confined |
| Q143 | 113.32 | 22.85 | 15.1 | Confined |
| Q130 | 113.24 | 23.06 | 13-15.8 | Confined |
| Q132 | 113.37 | 23.10 | 11-14.1 | Confined |
| Q124 | 113.41 | 23.13 | 18.8-24.8 | Confined |
| Q135 | 113.29 | 23.01 | 13.2 | Confined |
